# Intra-cerebellar schwannoma with various degenerative changes: a case report and a systematic review

**DOI:** 10.1186/s12883-022-02596-3

**Published:** 2022-02-24

**Authors:** Yasuhide Takeuchi, Yoshiki Arakawa, Hideaki Yokoo, Yoshiki Mikami, Yukinori Terada, Kazumichi Yoshida, Susumu Miyamoto, Hironori Haga

**Affiliations:** 1 Department of Diagnostic Pathology, Kyoto University Graduate School of Medicine, 54 Shogoin Kawahara-cho, Sakyo-ku, 606-8507 Kyoto, Japan; 2 Department of Neurosurgery, Kyoto University Graduate School of Medicine, 54 Shogoin Kawahara-cho, Sakyo-ku, Kyoto, 606-8507 Japan; 3 Department of Human Pathology, Gunma University Graduate School of Medicine, 3-39-22, Showa-machi, Gunma 371-8511 Maebashi City, Japan; 4 Department of Diagnostic Pathology, Kumamoto University Hospital, 1-1-1 Honjo, Chuo-ku, 860-8556 Kumamoto City, Japan

**Keywords:** Schwannoma with degenerative changes, Ancient schwannoma, Intra-cerebellar schwannoma, Eosinophilic granular inclusions

## Abstract

**Background:**

Intra-cranial schwannomas account for less than 8% of brain tumors, among which more than 80% arise from the vestibular nerve. Intra-cerebellar schwannomas are extremely rare. Several cases have been previously reported but without remarkable degenerative changes on histology.

**Case presentation:**

A 61-year-old man presented with worsening disorientation, and an imaging study revealed a cystic lesion (6.5 cm in the largest diameter) in the left hemisphere of the cerebellum accompanied by a mural nodule (2.5 cm) located just inside the skull with enhancement and focal calcification, in addition to hydrocephalus. The lesion was more than 5 mm from the left acoustic nerve. The patient underwent gross total resection. Pathological examination revealed remarkable degenerative changes with various morphological features. Tumor cells were pleomorphic with rich cytoplasm containing numerous eosinophilic granules. Blood vessels and extracellular matrix showed remarkable hyalinization. Immunohistochemical staining revealed that the tumor cells were positive for S-100 protein and negative for Olig2. The tumor was diagnosed as a schwannoma with marked degenerative changes.

**Conclusions:**

The present case is discussed with reference to a systematic review of previous reports of intra-cerebellar schwannoma. Intra-cerebellar schwannoma should be included in the differential diagnosis of cystic lesions with heterogeneous histopathological morphology in the cerebellum.

## Introduction

Schwannoma, also known as neurilemoma or neurinoma, is a benign nerve sheath tumor arising from differentiated Schwann cells [1, 2]. Intra-cranial schwannomas account for 5–8% of intra-cranial tumors [2], and approximately 90% of them arise in cerebellopontine angles in relation to the vestibular nerve. Sub-tentorial intra-cerebral schwannomas are very rare, and to date, only 20 cases of cerebellar schwannomas have been reported in the English literature [3–18]. In this report, we present a case of schwannoma, without a family history of neurofibromatosis, arising within the cerebellar hemisphere with remarkable degenerative changes on histology.

## Case presentation

**Fig. 1:**
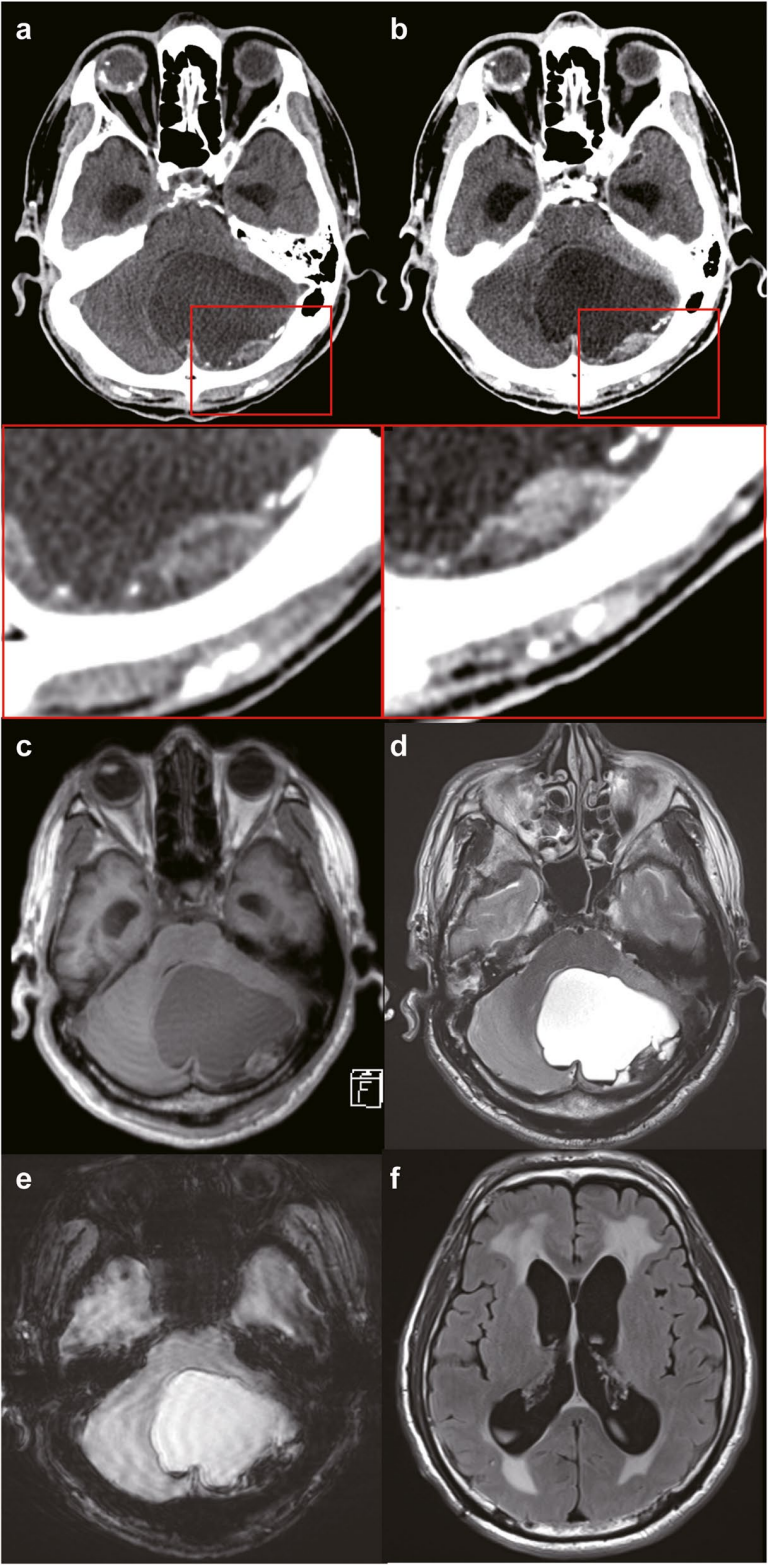
Pre-operative images of computed tomography (CT) scans and magnetic resonance imaging (MRI). **a** CT revealed a cystic lesion with a mural nodule in the left hemisphere of the cerebellum. The lower panel shows a magnified image of the area within the red rectangle in the upper panel. The nodular component was accompanied by a high attenuated area, suggesting focal calcification. Hydrocephalus was associated with a shift of the fourth ventricle to the right. **b** Contrast-enhanced CT showed mild enhancement in the mural nodule. The lower panel shows a magnified image of the area within the red rectangle in the upper panel. **c**, **d** MRI demonstrated that the cystic lesion was hypointense on a T1-weighted image (**c**) and hyperintense on a T2-weighted image (**d**). The tumor was well-defined, and no peritumoral brain edema was present. **e** T2 star-weighted image shows a hemorrhage within the cyst and in the mural nodule. **f** Fluid-attenuated inversion recovery image shows hydrocephalus with periventricular hyperintensity

**Fig. 2:**
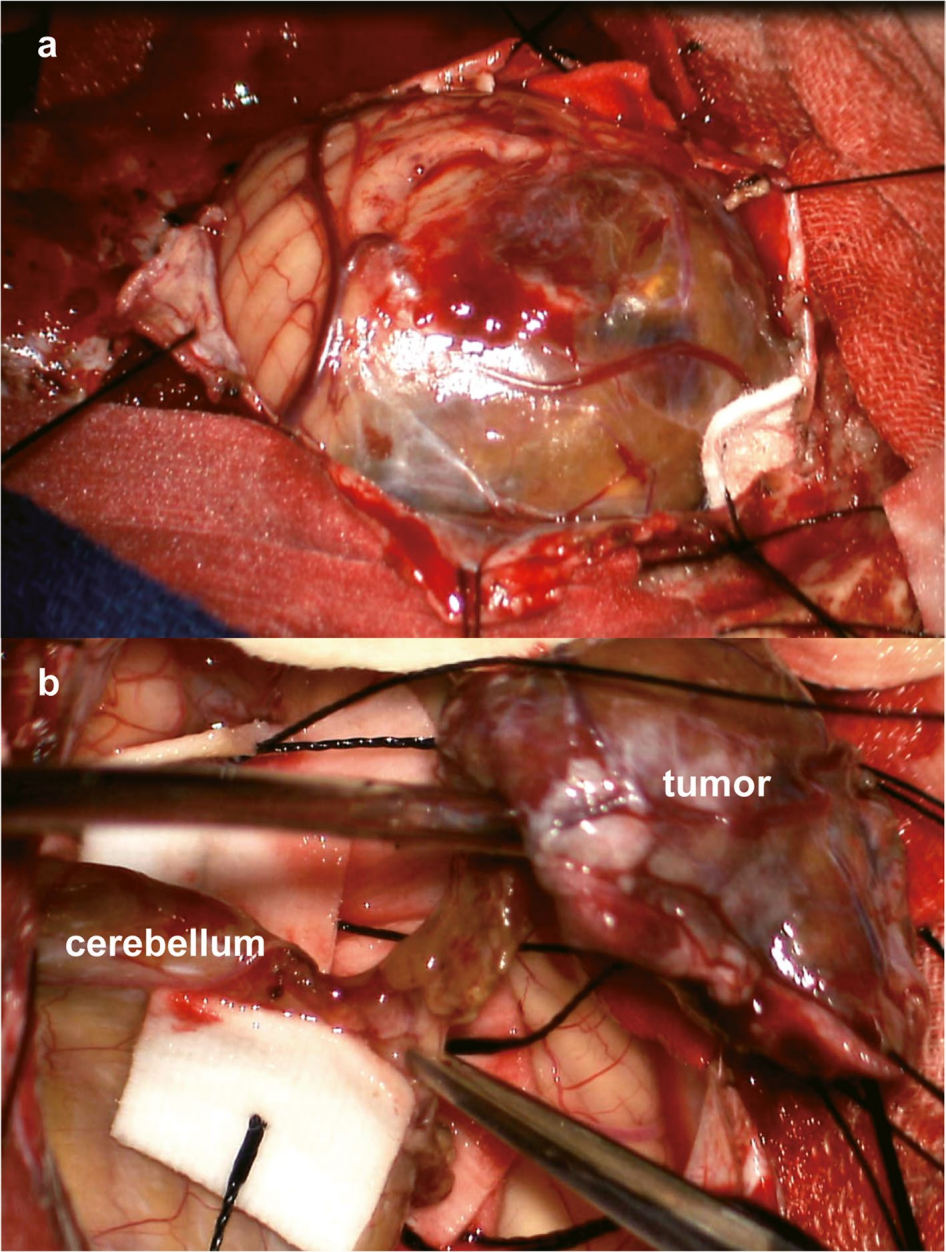
Macroscopic views of the cerebellar tumor. **a** The surface of the tumor was moderately swollen with a cystic component filled with brownish fluid, implying a history of intra-cystic bleeding. **b** The boundary of most of the tumor was clear. The tumor was resected totally after detachment from the cerebellum

A few days after amputation of the left lower leg because of arteriosclerosis obliterans, a 61-year-old man presented with worsening disorientation and was referred to our institution. He had been undergoing treatment for hypertension and diabetes mellitus for around a year and undergoing artificial dialysis due to chronic renal failure for 4 years. He had no symptoms and no family history of neurofibromatosis. Neurological examination demonstrated disorientation in date and time, and finger-nose-finger and heel-knee-shin dysmetria on the left side but no hearing disturbance. A computed tomography scan revealed a cystic lesion (6.5 cm in the largest diameter) in the left hemisphere of the cerebellum with a mural nodule (2.5 cm) with enhancement and focal calcification, in addition to hydrocephalus (Fig. 1a, b). Magnetic resonance imaging demonstrated that the cystic lesion was hypointense on T1-weighted images and hyperintense on T2-weighted images (Fig. 1c, d). The tumor was well defined, and no peritumoral brain edema was observed. The lesion was more than 5 mm from the left acoustic nerves, and the nodular component was located just inside the skull. T2 star-weighted images showed hemorrhage within the cyst and in the mural nodule (Fig. 1e). Fluid-attenuated inversion-recovery images showed hydrocephalus with periventricular hyperintensity (Fig. 1f). Pre-operative differential diagnoses included hemangioblastoma, low-grade well-circumscribed glioma, and metastatic tumor of unknown primary origin. The patient underwent suboccipital craniotomy. The surface of the tumor was moderately swollen (Fig. 2a). The discharge of brownish fluid during drainage of the cystic component implied a history of intra-cystic bleeding. The tumor had feeders from the superior cerebellar artery, and most of the boundary was clear. The tumor was resected totally after detachment from the cerebellum (Fig. 2b). The post-operative clinical course was good, and hydrocephalus improved. A 5-year follow-up demonstrated no recurrence or neurological dysfunction.

**Fig. 3:**
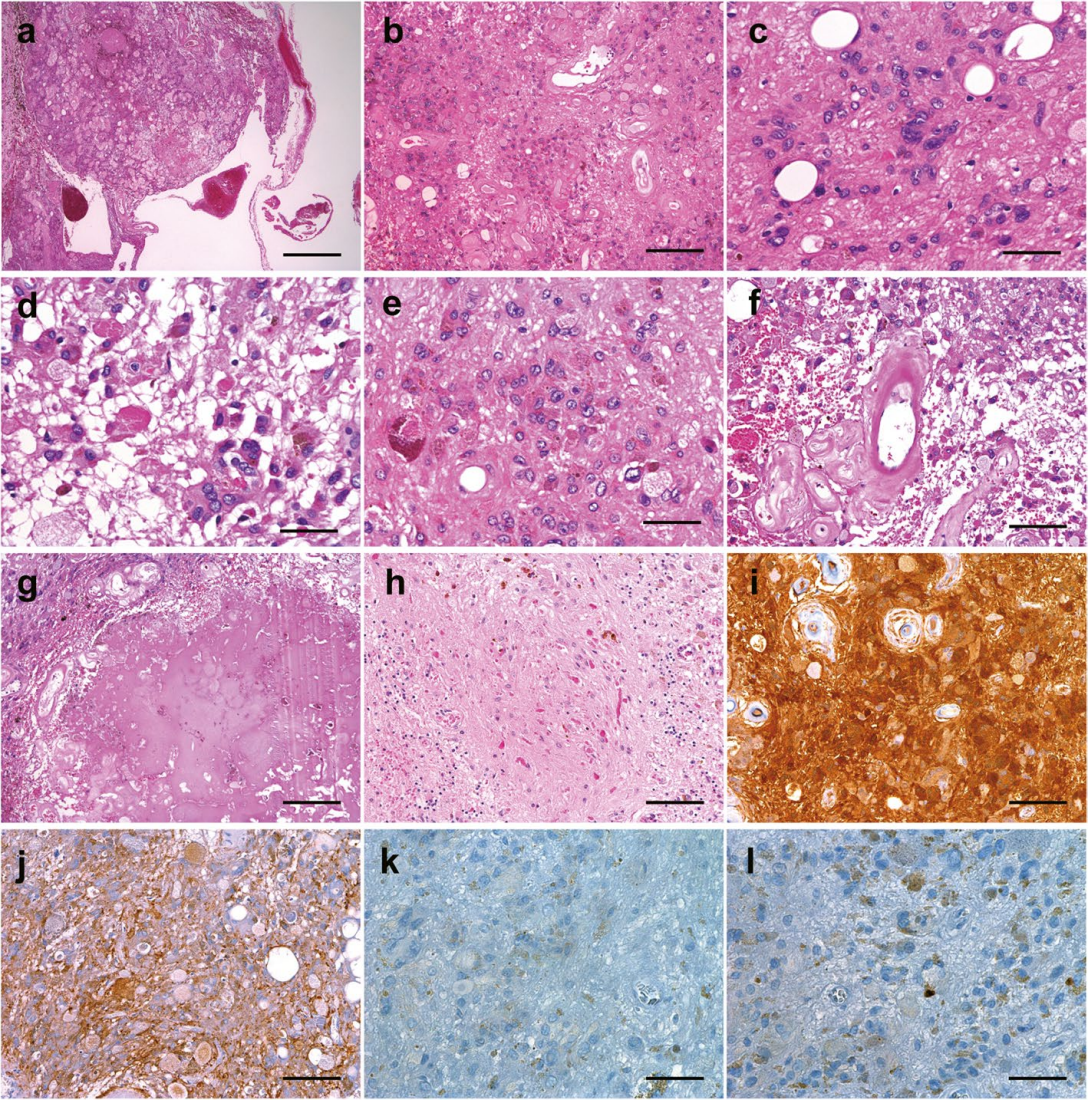
Histopathological examination of the excised tumor. **a** Low-power view of the excised tissue with hematoxylin and eosin staining. On the periphery of the tumor tissue, cystic components were observed. **b** Middle-power view of the tumor tissue. Tumor cells were intermingled with hyalinized blood vessels. **c**-**e** High-power view of the tumor cells. The tumor cells were pleomorphic and harbored eosinophilic cytoplasm and hyperchromatic nuclei (**c**). A meshwork of fine fibers was observed in the acellular area, and cells with eosinophilic granular inclusions or hyaline bodies were observed (**d**). Lipofuscin granules showed various colors, from faint pinkish to dark brown (**e**). **f**,** g** Hyalinization was diffusely observed in the wall of the blood vessels and intra-tumoral matrix. **h** Scattered Rosenthal fibers were observed in the cerebellum surrounding the tumor. **i**-**l** Representative images of immunohistochemical analyses. Tumor cells were diffusely positive for S-100 protein (**i**) and focally positive for glial fibrillary acidic protein (**j**). Tumor cells were negative for Olig2 (**k**). The Ki-67 labeling index was less than 1% (**l**). Scale bars: 1 mm (**a**), 200 μm (**b**,** g**), 100 μm (**f**,** h**-**j**), and 50 μm (**c**-**e**,** k**,** l**)

Histopathological examination of the tumor tissue showed proliferation of tumor cells with large nuclei intermingled with various sized blood vessels (Fig. 3a, b). The tumor cells were pleomorphic, with polygonal or spindle shapes, and harbored eosinophilic cytoplasm and hyperchromatic nuclei (Fig. 3c). The size of the nuclei also varied, and intra-nuclear cytoplasmic inclusions were occasionally observed. Nucleoli were occasionally observed and mostly inconspicuous. Cellular areas and hypo-cellular areas were intermingled, and a meshwork of fine fibers was observed in the hypo-cellular areas (Fig. 3d). Most tumor cells harbored abundant cytoplasm with a low nuclear-cytoplasmic ratio, and no mitosis was detected. Various intra-cytoplasmic inclusions were observed, including eosinophilic granular inclusions or hyaline bodies, concentric cytoplasmic inclusions, and yellow-brownish lipofuscin granules (Fig. 2d). The lipofuscin granules showed various colors, from faint pinkish to dark brown (Fig. 3e). Cells with eosinophilic granular inclusions or hyaline bodies tended to be distributed around hemosiderin-laden macrophages or hypo-cellular areas. Hyalinization was diffusely observed in the wall of the blood vessels and intra-tumoral extracellular matrix (Fig. 3f, g). On the periphery of the tumor, hemorrhage, aggregation of hemosiderin-laden macrophages, and cystic areas were observed (Fig. 3a). Calcification was occasionally observed. Neither Rosenthal fibers nor neuronal differentiation were observed within the tumor. The cerebellum surrounding the tumor showed reactive gliosis and mild atrophy, as well as scattered Rosenthal fibers (Fig. 3h). Immunohistochemical analyses showed that the tumor cells were diffusely positive for S-100 protein (Fig. 3i), vimentin, and neuron-specific enolase and focally positive for glial fibrillary acidic protein (Fig. 3j). Tumor cells were negative for Olig2 (Fig. 3k), synaptophysin, chromogranin, neurofilament, NeuN, epithelial membrane antigen, CD34, IDH1 (R132H), Melan A, and Inhibin alpha. The Ki-67 labeling index was less than 1%, with only a few positive cells (Fig. 3l). Considering various degenerative changes of the tumor tissue and the immunohistochemical features, the final pathological diagnosis of schwannoma with marked degenerative changes (a so-called ancient schwannoma) was made.

## Discussion and conclusions

Schwannomas arise from differentiated Schwann cells [1, 2], and are subdivided into variants based on histological features, including conventional or classic, cellular, ancient, plexiform, and melanotic. The ancient variant is the third most common form of schwannoma variants, and secondary degenerative changes are evident along with an extreme degree of hyalinization of the vascular wall [2]. In the present case, along with hyalinization of the blood vessels, eosinophilic granular inclusions or hyaline bodies were diffusely observed. Granular inclusions such as those observed in the present case were reported to be frequently observed in intra-cranial schwannomas, especially acoustic schwannomas [19, 20], as products of degeneration, and tend to be distributed around the periphery of the Antoni B pattern or area with rich hemosiderin deposition. Pilocytic astrocytoma (PA) should be considered in the differential diagnosis of cerebellar tumors with remarkable degenerative changes. PA is frequently observed in the cerebellum, and is also known to accompany degenerative cellular atypia, vascular hyalinization, calcification, and eosinophilic granular bodies [1]. Immunohistochemical staining for Olig2 is useful in differentiating PA and schwannoma; the former is usually positive, and the latter is not [21]. Although pre-operative differential diagnoses did not include schwannoma, these findings, in combination with immunohistochemical staining for the S-100 protein and Olig2, led to the diagnosis of an ancient schwannoma in the present case.

**Fig. 4:**
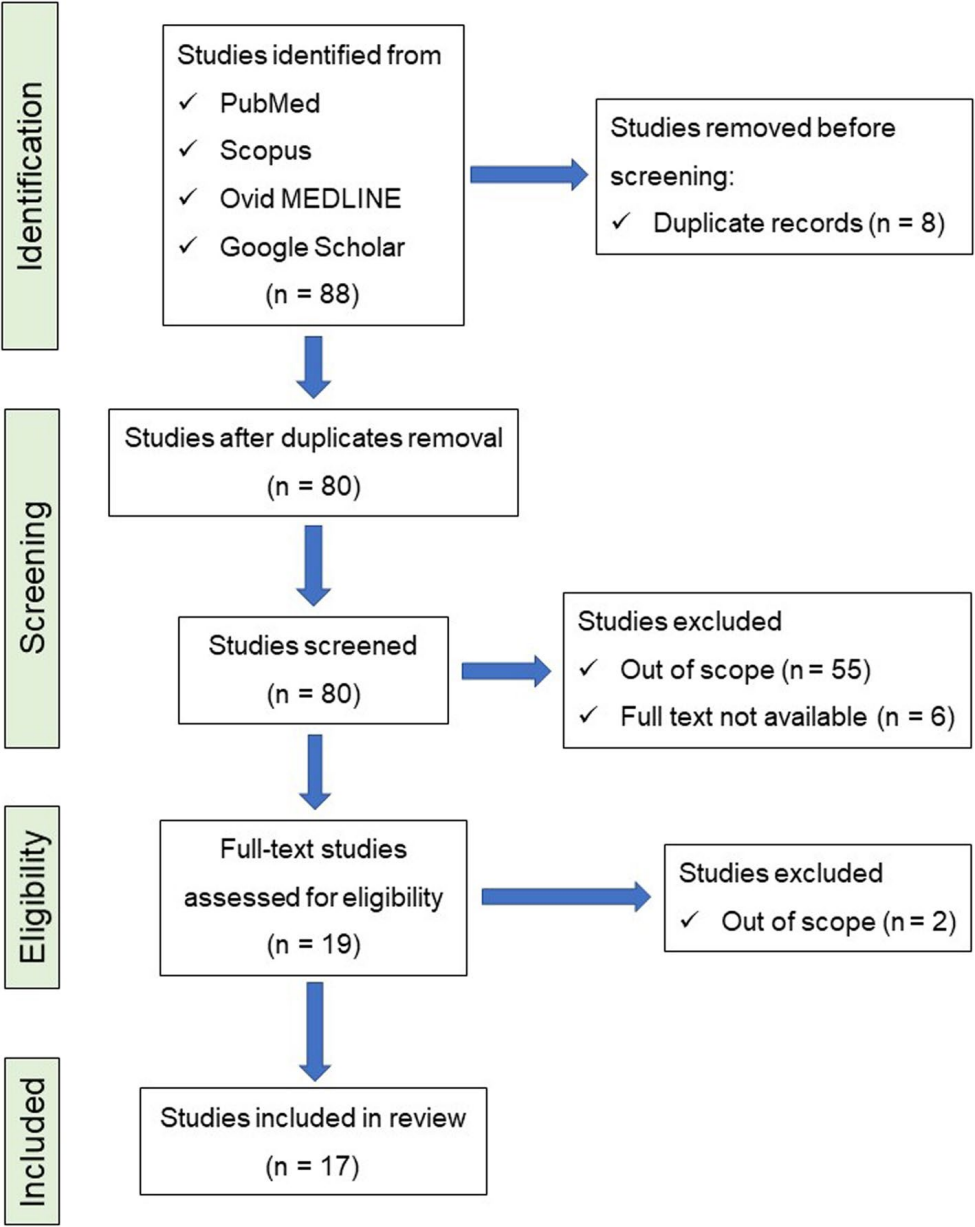
Flow diagram of systematic literature review

We aimed to identify all full-text, peer-reviewed publications describing the detailed clinical findings in intracerebellar schwannoma. A systematic review was performed according to the guidelines of the Preferred Reporting Items for Systematic Reviews and Meta Analyses (PRISMA) [22] (Fig. 4). Published studies pertaining to intracerebellar schwannoma were found by utilizing a thorough search strategy of PubMed, Scopus, Ovid MEDLINE, and Google Scholar databases from inception to February 1, 2022, with no language or regional restrictions. The term “Intracerebellar schwannoma” and “cerebellar schwannoma” was used for the search. The reference lists of chosen articles were searched to further identify relevant articles. The information that was extracted were as follows: author; year; clinical findings of the patients, including age, sex, tumor location, history of the diagnosis of neurofibromatosis, cystic change of the tumor detected by imaging study, dilation of ventricles and/or compression of ventricles. After removal of redundant reports, a total of 80 literatures were screened by the title/abstract, and 19 literatures were screened by full text. Finally a total of 17 eligible literatures with 20 cases were included to this study. To date, in combination with the present case, a total of 21 cases of cerebellar schwannoma have been reported in the English literature [3–18, 23]. The patient characteristics and findings are summarized in Table 1. Age at diagnosis ranged from 9–79 years old (y.o.) (Mean: 47.0 y.o., Median: 49.0 y.o.), and the ratio of male/female was 0.5 (7/14 patients, respectively). Distribution of the age of onset is similar to the cases of intra-cranial vestibular schwannomas described in a previous report, meanwhile the male/female ratio is lower [24]. Based on diagnostic imaging, 16 cases were described to be accompanied by cystic changes, which is a common feature of classical or ancient schwannomas [25]. Among 21 cases of cerebellar schwannomas, 11 tumors were located across the cerebellar vermis, and 13 cases were reported to harbor dilation of the lateral ventricles and/or compression of the fourth ventricle because of growth of the tumor or expansion of cystic components. In the present case, pathological examination revealed the slow proliferative nature of the tumor cells, with no mitotic figures, a low Ki-67 labeling index, and remarkable degenerative changes. The nodular component of the tumor was just inside the skull, and the cystic component extended into the cerebellar parenchyma and pressed on the fourth ventricle. The rapid progression of cognitive dysfunction in the patient may be explained by the rapid expansion of the cystic component of the tumor, resulting in hydrocephalus due to compression of the fourth ventricle and cerebellar parenchyma.

**Table 1:** Summary of previously reported cases of cerebellar schwannoma

Authors	Age years	Sex	Tumor location	Neurofibromatosis	Cystic change	Dilation of Ventricles and/or compression of ventricles	Ref
Kuhn et al., 1985	42	F	Cerebellar vermis	N. D	Yes	N. D	[8]
Sarkar et al., 1987	24	M	Cerebellum, just left to the midline	N. D	Yes	Yes	[11]
Schwartz and Sotrel, 1988	48	M	Cerebellar hemisphere	No	Yes	Yes	[12]
Tran-Dinh et al., 1991	64	F	Cerebellar vermis	N. D	Yes	Yes	[15]
Chitre et al., 1992	35	F	Cerebellar vermis	No	Yes	Yes	[5]
Casadei et al., 1993	52	F	Cerebellar hemisphere	No	Yes	N. D	[4]
Casadei et al., 1993	55	M	Cerebellar hemisphere	No	Yes	N. D	[4]
Casadei et al., 1993	79	F	Cerebellar vermis	No	Yes	N. D	[4]
Sharma et al., 1993	73	F	Cerebellar vermis to cerebellar hemisphere	No	N. D	Yes	[23]
Sharma et al., 1996	45	M	Cerebellar vermis and crossing midline	No	N. D	N. D	[13]
Sharma et al., 1996	24	M	Cerebellum, NOS	No	N. D	N. D	[13]
Ranjan et al., 1995	65	F	Cerebellar hemisphere	N. D	No	Yes	[10]
Tanabe et al., 1996	68	F	Cerebellar hemisphere	No	Yes	Yes	[14]
Tsuiki et al., 1997	64	F	Cerebellar hemisphere	No	Yes	N. D	[16]
Bjatjiwale and Gupta, 1999	15	M	Cerebellar vermis	No	Yes	Yes	[3]
Jabbour et al., 2002	9	F	Cerebellar hemisphere	No	Yes	Yes	[7]
Maiuri et al., 2004	29	F	Cerebellar vermis	No	N. D	Yes	[9]
Chung et al., 2007	49	F	Cerebellar hemisphere	No	Yes	Yes	[6]
Umredkar et al., 2011	35	F	Cerebellar vermis	No	Yes	Yes	[17]
Xuejian et al., 2013	52	F	Cerebellar hemisphere	No	Yes	N. D	[18]
Present Case	61	M	Cerebellar hemisphere to cerebellar vermis	No	Yes	Yes	-

*N.D*. Not Described, *Ref* Reference number

The origin of intra-cerebellar schwannomas remains unclear. Some researchers suggest that the origin of intra-cerebellar schwannomas is Schwann cells in the perivascular plexus [15], and others suggest that specific soft membrane cells undergo metaplastic changes into Schwann cell-like cells and result in the formation of a schwannoma [26]. Either hypothesis can explain the pathogenesis of the schwannoma in the present case, and rapid expansion of the cystic component may make analysis of the pathogenesis difficult, because of destruction of the surrounding structures. The distribution and dynamics of the stem cells in the central nervous system has still not been fully elucidated [27]. To fully understand the pathogenesis of the intra-cerebellar schwannomas, detailed morphological analysis of the intact tumor and surrounding tissue is mandatory.

In this report, we presented a case of schwannoma arising within cerebellar hemisphere with remarkable degenerative changes. Although it’ rare, schwannoma should be listed in the differential diagnosis of the cystic lesion with heterogeneous histopathological morphology in the cerebellum.

## Data Availability

Not applicable.

## Abbreviations

CT: Computed tomography
MRI: Magnetic resonance imaging
FLAIR: Fluid-attenuated inversion-recovery
NSE: Neuron-specific enolase
GFAP: Glial fibrillary acidic protein
EMA: Epithelial membrane antigen
